# Aberrant phenotype and transcriptome expression during fiber cell wall thickening caused by the mutation of the *Im* gene in immature fiber (*im*) mutant in *Gossypium hirsutum* L

**DOI:** 10.1186/1471-2164-15-94

**Published:** 2014-02-01

**Authors:** Cheng Wang, Yuanda Lv, Wentin Xu, Tianzhen Zhang, Wangzhen Guo

**Affiliations:** 1 State Key Laboratory of Crop Genetics & Germplasm Enhancement, Hybrid Cotton R & D Engineering Research Center, MOE, Nanjing Agricultural University, Nanjing 210095, China

**Keywords:** Immature fiber mutant, Expression profiling, Fiber secondary cell wall thickening, Fiber micronaire, Microarray, *Gossypium hirsutum*

## Abstract

**Background:**

The immature fiber (*im*) mutant of *Gossypium hirsutum* L. is a special cotton fiber mutant with non-fluffy fibers. It has low dry weight and fineness of fibers due to developmental defects in fiber secondary cell wall (SCW).

**Results:**

We compared the cellulose content in fibers, thickness of fiber cell wall and fiber transcriptional profiling during SCW development in *im* mutant and its near-isogenic wild-type line (NIL) TM-1. The *im* mutant had lower cellulose content and thinner cell walls than TM-1 at same fiber developmental stage. During 25 ~ 35 day post-anthesis (DPA), sucrose content, an important carbon source for cellulose synthesis, was also significantly lower in *im* mutant than in TM-1. Comparative analysis of fiber transcriptional profiling from 13 ~ 25 DPA indicated that the largest transcriptional variations between the two lines occurred at the onset of SCW development. TM-1 began SCW biosynthesis approximately at 16 DPA, whereas the same fiber developmental program in *im* mutant was delayed until 19 DPA, suggesting an asynchronous fiber developmental program between TM-1 and *im* mutant. Functional classification and enrichment analysis of differentially expressed genes (DEGs) between the two NILs indicated that genes associated with biological processes related to cellulose synthesis, secondary cell wall biogenesis, cell wall thickening and sucrose metabolism, respectively, were significantly up-regulated in TM-1. Twelve genes related to carbohydrate metabolism were validated by quantitative reverse transcription PCR (qRT-PCR) and confirmed a temporal difference at the earlier transition and SCW biosynthesis stages of fiber development between TM-1 and *im* mutant.

**Conclusions:**

We propose that *Im* is an important regulatory gene influencing temporal differences in expression of genes related to fiber SCW biosynthesis. This study lays a foundation for cloning the *Im* gene, elucidating molecular mechanism of fiber SCW development and further genetic manipulation for the improvement of fiber fineness and maturity.

## Background

Cotton (*Gossypium* sp.) is an important economic crop, as it is the leading natural textile fiber used worldwide. Each cotton fiber is a unicellular hair originating from the seed epidermis. Fiber development consists of four overlapping stages: fiber initiation, fiber elongation, secondary cell wall (SCW) deposition and maturation. Fiber initiation occurs on or near the day of anthesis, but only about 25% of ovular epidermal cells differentiate into the commercially important and spinnable lint fibers. Fibers rapidly elongate during 5 ~ 20 days post-anthesis (DPA) with peak growth rates of >2 mm/day. At the late stage of fiber elongation, fibers enter into SCW deposition. The onset of SCW biosynthesis typically occurs from 16 ~ 21 DPA, depending on the cotton species and environmental conditions. Successive fiber developmental stages overlap by several days. During SCW biosynthesis, a large amount of cellulose is synthesized, and this process persists for a long period of time until fiber maturation. When bolls open at ~50 DPA, fibers quickly dehydrate and then become mature. Greater than 90% of the dry weight of the mature fibre exists as cellulose [1,2]. Because of the unique characteristics of cotton fiber, it is regarded as an ideal model for studies of plant cell elongation and cell wall biogenesis [3].

Mutants are powerful tools for studies of molecular mechanisms of important genes. Some key cotton fiber mutants have been obtained, such as the *Ligon Lintless* mutant *(Li1* and *Li2)*, *naked seed* mutant *(N1* and *n2)* and *fuzzless-lintless* mutant *(fl).* However, all of these fiber mutants are defective in fiber initiation or elongation. Kohel and McMichael [4] first reported the *immature fiber* mutant (*im*), which has completely different characteristics from those mutants described above. After the bolls open, fibers in the *im* mutant are matted around the seed and are not as fluffy as those observed in normal cotton lines. The fiber phenotype in *im* mutant is similar to the appearance of cotton bolls in normal plants subjected to stressors, such as moisture, low temperature or disease. Additionally, the *im* mutant has low dry weight and fineness of fibers, reflecting defects in development [4]. The *im* locus is located on chromosome 3 [5,6], and it was recently finely mapped almost simultaneously by Wang et al. [7] and Kim et al. [8].

The transcriptome of cotton fiber is extraordinarily complex, involving varied expression of tens of thousands of genes. In order to understand fiber development better, it is important to study the expression patterns of relevant genes and define multi-gene interactions. Gene chip technology provides a powerful approach for simultaneously analyzing differential expression of genes in a high throughput manner. Expression profiling of cotton using microarrays has been the subject of several recent studies on functions of key genes [9-11], development [10,12-14], genome evolution [15-18] and responses to stress [19-21].

In this study, significant differences in cellulose content, thickness of fiber cell wall and the content of several soluble carbohydrates were revealed through comparing the *im* mutant with its near-isogenic line (NIL) TM-1 during fiber development. We performed a comparative analysis of transcriptional profiles between TM-1 and the *im* mutant during fiber SCW development using cDNA microarrays, identified and analyzed differentially expressed genes (DEGs) and their potential functional roles during fiber development. This study establishes a foundation for elucidating the molecular mechanism of the mutated gene and genetic manipulation for the improvement of fiber fineness and maturity.

## Results

### Decreased cellulose content and thickness of fiber cell wall in *im* mutant

**Figure 1:**
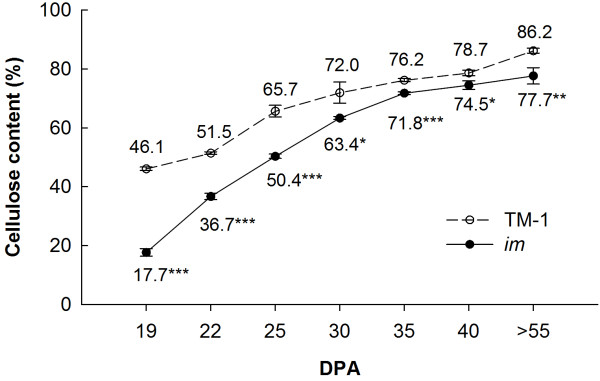
**Cellulose content in fibers in TM-1 and *im *mutant.** Each value is the mean ± SD of three biological replicates. Asterisks indicate significant differences (Student’s *t* test, *P < 0.05, **P < 0.01, ***P < 0.001) between TM-1 and the *im* mutant at same developmental stage.

In order to explore possible physiological changes in *im* mutant caused by the mutation of the *Im* gene, we compared the cellulose content, a major cell wall component of SCW in fibers between the *im* mutant and its near-isogenic line (NIL), TM-1, during SCW thickening (Figure 1). Cellulose content in the *im* mutant was significantly lower than that in TM-1 at each surveyed fiber developmental time point. However, a relatively larger difference was observed during the early stages of SCW development, and the cellulose of mature fibers was decreased by only about 10% in the *im* mutant, indicating that the two lines had different cellulose synthesis patterns and that the *Im* gene may have had more influence on fiber development at early stages of SCW synthesis.

**Figure 2:**
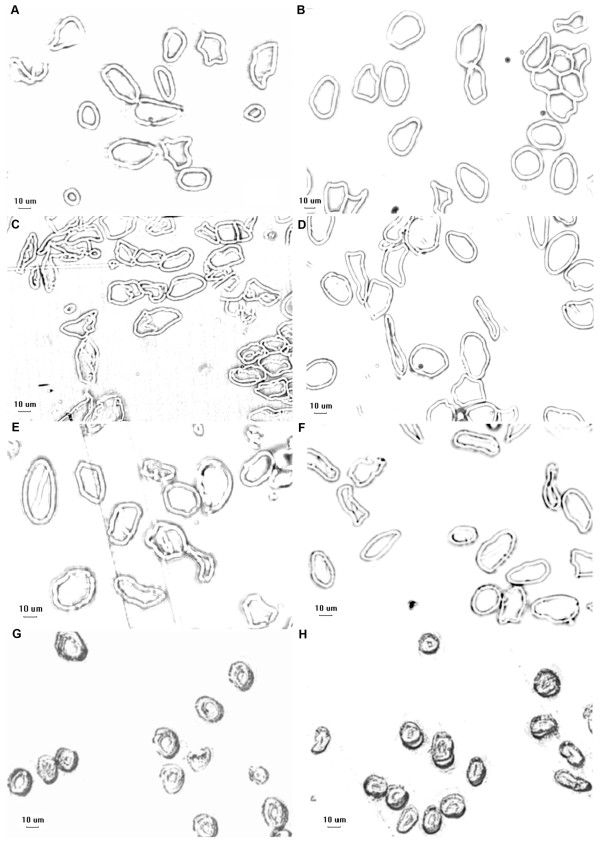
**Cell wall thickness of fibers in TM-1 and *im *mutant. A**, *im* 13 DPA; **B**, TM-1 13 DPA; **C**, *im* 19 DPA; **D**, TM-1 19 DPA; **E**, *im* 25 DPA; **F**, TM-1 25 DPA; **G**, *im* > 55 DPA; **H**, TM-1 > 55 DPA. Bar = 10 μm. Statistical analysis result is listed in Table 1.

To explore the effect of reduced cellulose on fiber development, fiber cell wall thickness was measured by microscopy at 13, 19, 25 DPA and maturity in TM-1 and the *im* mutant plants (Figure 2 and Table 1). There was no difference between the two lines at 13 and 19 DPA, but the *im* mutant had thinner fiber cell walls (*P* < 0.001) than TM-1 at 25 DPA and maturity (stages of rapid cellulose synthesis and highest cellulose content, respectively). These results suggest that the developmental defect in the fiber SCW and production of thinner fiber cell wall in the *im* mutant was most likely due to the reduction of cellulose.

**Table 1:** **Difference of thickness of fiber cell wall between TM-1 and *im *** **mutant**

**DPA**	**TM-1 (μm)**	***im *** **(μm)**	**t value**	***P *** **value**
13	3.13 ± 0.18	3.12 ± 0.20	1.23	0.221
19	3.16 ± 0.20	3.16 ± 0.15	0.093	0.926
25	3.31 ± 0.17	3.21 ± 0.19***	7.25	1.21E-12
>55	5.97 ± 0.83	4.77 ± 0.58***	20.03	3.07E-66

The thickness of fiber cell wall showed in Figure 2. Approximately 300 fibers were measured for each sample. Each value is the mean ± SD.

***P < 0.001 (Student’s *t* test).

### Sucrose content is significantly different during fiber SCW development between *im* mutant and TM-1

**Figure 3:**
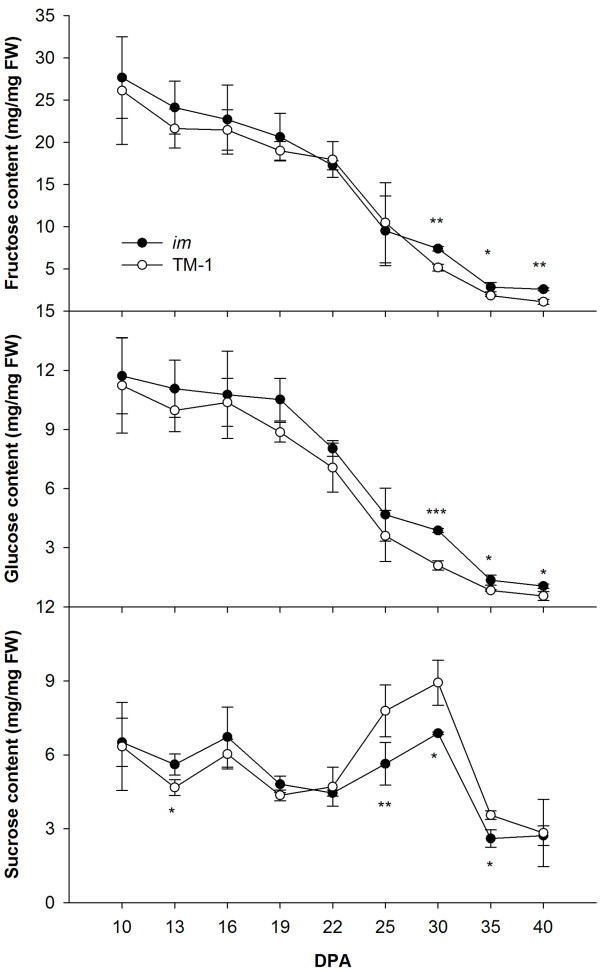
**Soluble carbohydrate content in fibers in TM-1 and *im *mutant.** Each value is the mean ± SD of three biological replicates. Asterisks indicate significant differences (Student’s *t* test, *P < 0.05, **P < 0.01, ***P < 0.001) between TM-1 and the *im* mutant at same developmental stage.

Cellulose synthesis requires consumption of a large amount of carbon source, mainly sucrose. Fructose and glucose are key substrates in the sucrose metabolic pathway, and they also participate in many important biological processes. Therefore, the fructose, glucose and sucrose contents in fibers of the *im* mutant and TM-1 were measured at 10, 13, 16, 19, 22, 25, 30, 35 and 40 DPA by high-performance liquid chromatography (HPLC) (Figure 3). TM-1 and *im* mutant plants showed similar changes in sugar content, and no difference in fructose and glucose content was observed between the two lines until the later stages of SCW development. The *im* mutant had higher fructose and glucose content during 30 ~ 40 DPA (*P* < 0.05). However, relatively large differences in sucrose content between TM-1 and the *im* mutant appeared from 22 DPA. The sucrose content in fibers of TM-1 was higher than that in the *im* mutant during the developmental interval 25 ~ 35 DPA, which corresponded to the difference in cellulose content between the two accessions. The normal carbohydrate metabolism was presumed to be affected by the mutation of the *Im* gene, and the level of sucrose available for the cellulose synthesis pathway in the *im* mutant was lower than that in TM-1.

### Differential gene expression in fibers between TM-1 and *im* mutant

**Figure 4:**
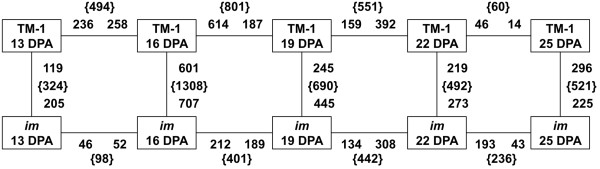
**Number of DEGs within and between TM-1 and *im *mutant.** Microarray analysis was performed on three replicates of each sample at five stages (represented by boxes) for both accessions. Numbers on or beside the line designate the number of up-regulated genes (at least 2-fold and FDR < 0.05) relative to their adjacent developmental stage or between two accessions, with the total number of genes in the comparison given in brackets.

To explore the impact of the mutation of the *Im* gene on global patterns of gene expression in developing fibers, gene expression levels in samples derived from the wild-type TM-1 and *im* mutant lines were compared at five fiber developmental stages (13, 16, 19, 22 and 25 DPA), and numbers of differentially expressed genes (DEGs) between adjacent time points during fiber development within and between the two lines were determined (Figure 4). The distribution of DEGs with a false discovery rate (FDR) < 0.05 and fold change ≥ 2 was roughly similar within TM-1 and *im* mutant, but the level of transcriptional variation between adjacent time points within the two lines was different. Far more genes showed altered expression (16 vs. 13 DPA and 19 vs. 16 DPA) in TM-1, compared to those in *im* mutant during the same fiber developmental interval. Conversely, more genes were differentially expressed in *im* mutant than in TM-1 between 22–25 DPA. Additionally, during the interval between 19–22 DPA, the number of DEGs within each accession became smaller. In TM-1, the maximum number of DEGs was found between the two adjacent time points of 16 and 19 DPA. Out of 801 DEGs, only 187 genes remained up-regulated at 19 DPA, whereas 614 genes were up-regulated at 16 DPA but down-regulated at 19 DPA, implying that 16 DPA is an important fiber developmental stage for TM-1. In the *im* mutant, only 98 genes (16 vs. 13 DPA) were differentially expressed, compared with 494 DEGs (16 vs. 13 DPA) in TM-1, indicating that the gene expression profiles at 13 and 16 DPA in the *im* mutant were very similar and that relatively few changes in gene expression occurred in the mutant during the fiber elongation period.

Differences in gene expression between TM-1 and the *im* mutant at each fiber developmental time point were also determined in order to reveal the transcriptomic changes associated with the mutation of the *Im* gene. Notably, the greatest number of varied genes between TM-1 and the *im* mutant was observed at 16 DPA, with a total of 1308 DEGs, suggesting that 16 DPA is a key developmental transition stage affected by the mutation of the *Im* gene.

### Gene ontology (GO) categories associated with DEGs within TM-1 and *im* mutant

In order to explore the functions associated with altered gene expression, we compiled DEGs for various comparisons of the array data into GO categories and used Fisher’s exact test to identify enriched GO terms. Enrichment analysis revealed that many over-represented GO terms (FDR < 0.05) were parent-child relationships. Therefore, we used the “show only most specific terms” function in the Blast2GO program to return the child GO terms or tip GO terms. The most specific GO terms in the biological process category between adjacent time points within TM-1 and the *im* mutant are listed in Table 2 and discussed below. The complete information on over-represented GO terms is presented in Additional file 1.

**Table 2:** **Catalog of specific biological processes statistically over-represented during fiber developmental stages of TM-1 and *im *** **mutant (FDR < 0.05)**

**Developmental stage comparison**	**Process upregulated in TM-1 or *im***	**GO annotation**	**Functional categories**	**FDR**
**16 vs. 13 DPA, up-regulated at 16 DPA**	**TM-1**	GO:0009834	Secondary cell wall biogenesis	1.7E-04
		GO:0010417	Glucuronoxylan biosynthetic process	2.7E-04
	***im***	NA	NA	NA
**16 vs. 13 dpa, up-regulated at 13 DPA**	**TM-1**	GO:0009813	Flavonoid biosynthetic process	7.1E-10
		GO:0010466	Negative regulation of peptidase activity	3.1E-02
	***im***	GO:0043617	Cellular response to sucrose starvation	8.9E-04
		GO:0006529	Asparagine biosynthetic process	9.3E-04
		GO:0009744	Response to sucrose stimulus	9.8E-03
		GO:0009646	Response to absence of light	9.8E-03
		GO:0009063	Cellular amino acid catabolic process	1.2E-02
		GO:0009750	Response to fructose stimulus	1.7E-02
		GO:0006541	Glutamine metabolic process	2.2E-02
		GO:0009749	Response to glucose stimulus	3.1E-02
		GO:0009835	Ripening	3.3E-02
**19 vs. 16 DPA, up-regulated at 19 DPA**	**TM-1**	NA	NA	NA
	***im***	GO:0009834	Secondary cell wall biogenesis	1.3E-03
		GO:0030244	Cellulose biosynthetic process	6.0E-03
		GO:0007047	Cellular cell wall organization	7.9E-03
		GO:0010417	Glucuronoxylan biosynthetic process	4.1E-02
**19 vs. 16 DPA, up-regulated at 16 DPA**	**TM-1**	GO:0010025	Wax biosynthetic process	7.7E-05
		GO:0009664	Plant-type cell wall organization	1.6E-02
		GO:0006723	Cuticle hydrocarbon biosynthetic process	1.6E-02
		GO:0009826	Unidimensional cell growth	3.1E-02
	***im***	GO:0043086	Negative regulation of catalytic activity	2.2E-02
**22 vs. 19 DPA, up-regulated at 22 DPA**	**TM-1**	GO:0010200	Response to chitin	2.0E-05
		GO:0050832	Defense response to fungus	6.1E-03
		GO:0009738	Abscisic acid mediated signaling pathway	2.8E-02
	***im***	GO:0010200	Response to chitin	9.0E-04
		GO:0050832	Defense response to fungus	2.9E-02
		GO:0001666	Response to hypoxia	3.7E-02
**22 vs. 19 DPA, up-regulated at 19 DPA**	**TM-1**	GO:0009834	Secondary cell wall biogenesis	2.2E-03
		GO:0016126	Sterol biosynthetic process	1.8E-02
		GO:0035435	Phosphate transmembrane transport	2.6E-02
	***im***	GO:0010025	Wax biosynthetic process	1.9E-08
		GO:0000038	Very-long-chain fatty acid metabolic process	1.3E-04
		GO:0035435	Phosphate transmembrane transport	1.0E-02
		GO:0006723	Cuticle hydrocarbon biosynthetic process	4.3E-02
**25 vs. 22 DPA, up-regulated at 25 DPA**	**TM-1**	GO:0043617	Cellular response to sucrose starvation	1.5E-02
		GO:0006529	Asparagine biosynthetic process	1.5E-02
	***im***	NA	NA	NA
**25 vs. 22 DPA, up-regulated at 22 dpa**	**TM-1**	GO:0009809	Lignin biosynthetic process	1.8E-03
	***im***	GO:0010200	Response to chitin	8.0E-17
		GO:0010120	Camalexin biosynthetic process	2.3E-02
		GO:0050832	Defense response to fungus	4.0E-02
		GO:0000169	Activation of MAPK activity involved in osmosensory signaling pathway	4.9E-02

NA, no result.

FDR, false discovery rate.

During the interval 13–16 DPA, up-regulated DEGs at 16 DPA in TM-1 were involved in “secondary cell wall biogenesis” (GO:0009834, FDR = 1.7E-4) and “glucuronoxylan biosynthetic process” (GO:0010417, FDR = 2.7E-4). The two over-represented GO terms contained some important SCW-related genes reported in previous studies, such as fasciclin-like arabinogalactan protein genes, *GhFLA5* and *GhFLA6*, which are putative homologues of *AtFLA12* and *AtFLA11*, respectively, previously implicated in SCW formation in *Arabidopsis thaliana*[22,23]; putative *PARVUS* gene which affects the development of SCW by regulating the biosynthesis of xylan [24]; and putative homologues of *Arabidopsis IRX7/FRA8*, *IRX9* and *IRX10/GUT2* genes necessary for normal SCW development [25-27]. In addition, *GhCesA2*, an important marker for onset of SCW biosynthesis gene in cotton fiber [28], and the *Chitinase-like* gene (*GhCTL2*), which is preferentially expressed during fiber SCW deposition, also showed higher transcript levels at 16 DPA [29]. Furthermore, no over-represented GO terms were among the up-regulated DEGs at 19 DPA in TM-1 (19 vs. 16 DPA). These results further indicated that onset of SCW biosynthesis for fiber in TM-1 occurred at 16 DPA.

During the same developmental interval 13–16 DPA, no GO terms (FDR < 0.05) were significantly up-regulated at 16 DPA in the *im* mutant. However, up-regulated DEGs at 19 DPA during the interval 16–19 DPA in *im* mutant were found to be involved in four biological processes associated with fiber cell wall development, including “cellular cell wall organization” (GO:0007047, FDR = 7.9E-3), “glucuronoxylan biosynthetic process” (GO:0010417, FDR = 4.1E-2), “secondary cell wall biogenesis” (GO:0009834, FDR = 1.3E-3) and “cellulose biosynthetic process” (GO:0030244, FDR = 6.0E-3). The results indicated that fibers in *im* mutant were undergoing cellulose synthesis and SCW thickening at 19 DPA, suggesting that SCW biosynthesis for fibers in *im* mutant began later (~19 DPA) than that in TM-1.

In TM-1, over-represented GO terms for up-regulated DEGs at 16 DPA during the interval 16-19 DPA included (i) “wax biosynthetic process” (GO:0010025, FDR = 7.7E-5), containing 3-ketoacyl-CoA synthase genes which participate in synthesis of very long-chain fatty acids and regulate fiber elongation [30]; (ii) “cuticle hydrocarbon biosynthetic process” (GO:0006723, FDR = 1.6E-2), containing the genes encoding WAX2 protein which influences the morphogenesis of trichomes [31]; (iii) “plant-type cell wall organization” (GO:0009664, FDR = 1.6E-2), containing expansin genes which play important roles in cell wall loosening and extension during early fiber development [32]; (iv) and “unidimensional cell growth” (GO:0009826, FDR = 3.1E-2), potentially reflecting the developmental process of polar cell elongation. In contrast, similar results were observed in the subsequent developmental comparison (22 vs. 19 DPA) in *im* mutant, and the same over-represented GO terms were up-regulated at 19 DPA (Table 2). These results provided indirect evidence that the fiber developmental program of secondary wall biosynthesis started earlier in TM-1 than in *im* mutant.

During the interval 19–22 DPA, TM-1 and *im* mutant had some over-represented GO terms in common for up-regulated DEGs at 22 DPA, suggesting that the two lines may have undergone similar biological processes during this stage of fiber development. However, over-represented GO terms unique to each of the two accessions were also found. For example, “abscisic acid mediated signaling pathway” (GO:0009738, FDR = 2.8E-2) was over-represented in TM-1, which is known to mediate fiber SCW thickening [33,34]. However, the over-represented GO terms specific to the *im* mutant were not associated with fiber development.

During the interval 22–25 DPA, over-represented GO terms among up-regulated genes at 25 DPA in TM-1 included “cellular response to sucrose starvation” (GO:0043617, FDR = 1.5E-2) and “asparagines metabolic process” (GO:0006528, FDR = 1.5E-2), which were potentially related to sucrose flux, an important sugar signaling for plant growth, development and physiology [35]. “Lignin metabolic process” (GO:0009808, FDR = 5.0E-3) was detected in the up-regulated genes at 22 DPA in TM-1. However, unlike SCWs of other plant cells, the cotton fiber SCW contains few non-cellulosic components and apparently little or no lignin. Genes in the lignin biosynthesis pathway may have other functions, since they also participate in other metabolic pathways, such as the phenylpropanoid pathway, and they have a potential effect on cotton fiber quality properties [11]. While no enriched GO terms for up-regulated DEGs was identified at 25 DPA in *im* mutant, “activation of MAPK activity involved in osmosensory signaling pathway” (GO:0000169, FDR = 4.9E-2) was over-represented among up-regulated DEGs at 22 DPA, reflecting the variation of cell osmotic potential in *im* mutant during fiber development.

### Over-represented GO terms of DEGs between TM-1 and *im* mutant during fiber development

Functional annotation and enrichment analysis of DEGs between TM-1 and *im* mutant at each time point were also performed by using the Blast2GO program. As described above, the most specific GO terms in biological process between the two lines are listed in Table 3 and discussed in the sections below. The complete information on over-represented GO terms is given in Additional file 2.

**Table 3:** **List of developmental stage-specific biological processes over-represented (FDR < 0.05) for up-regulated DEGs in TM-1 (TM-1 vs. *im *** **mutant)**

**Developmental stage**	**GO annotation**	**Functional categories**	**FDR**
**13 DPA**	GO:0010200	Response to chitin	6.8E-08
	GO:0050832	Defense response to fungus	3.9E-02
**16 DPA**	GO:0009086	Methionine biosynthetic process	8.0E-06
	GO:0030244	Cellulose biosynthetic process	9.9E-04
	GO:0009834	Secondary cell wall biogenesis	3.2E-03
	GO:0010417	Glucuronoxylan biosynthetic process	4.1E-03
	GO:0006529	Asparagine biosynthetic process	4.1E-03
	GO:0010248	Establishment or maintenance of transmembrane electrochemical gradient	5.4E-03
	GO:0010951	Negative regulation of endopeptidase activity	5.4E-03
	GO:0015992	Proton transport	1.4E-02
	GO:0043617	Cellular response to sucrose starvation	2.0E-02
	GO:0007047	Cellular cell wall organization	2.4E-02
	GO:0019305	dTDP-rhamnose biosynthetic process	3.2E-02
**19 DPA**	GO:0005985	Sucrose metabolic process	2.8E-04
	GO:0010417	Glucuronoxylan biosynthetic process	9.3E-03
	GO:0009834	Secondary cell wall biogenesis	2.5E-02
	GO:0009877	Nodulation	3.5E-02
	GO:0052386	Cell wall thickening	3.7E-02
**22 DPA**	NA	NA	NA
**25 DPA**	GO:0010200	Response to chitin	5.2E-28
	GO:0050832	Defense response to fungus	2.4E-04
	GO:0006351	Transcription, DNA-dependent	2.5E-03
	GO:0009725	Response to hormone stimulus	5.2E-03
	GO:0045087	Innate immune response	4.1E-02
	GO:0009409	Response to cold	4.3E-02

NA, no result.

FDR, false discovery rate.

A comparison of DEGs in the two lines at 13 DPA showed “response to chitin” (GO:0010200, FDR = 6.8E-8) and “defense response to fungus” (GO:0050832, FDR = 3.9E-2) were over-represented (FDR < 0.05). Chitin is a common component of fungal cell walls. When fungi attack, plant immune responses are induced, and an enzyme that digests chitin is produced [36]. However, cotton fibers are unlikely to be primary target sites of attack by fungi. Many of the DEGs with enriched GO terms encode transcription factors (TFs), such as zinc finger protein (*ATL2*, *CZF1/ZFAR1*), heat shock factor (*HSFB2A*), AP2/ERF (*ERF2*) and WRKY (*WRKY33*). TFs generally have multiple functions in diverse processes. Therefore, DEGs related to these over-represented GO terms may play other roles in the fiber development process and require further investigation.

At 16 DPA, the following biological processes for up-regulated DEGs in TM-1 were included in the over-represented categories: (i) “secondary cell wall biogenesis” (GO:0009834, FDR = 3.2E-3), the most important event for fiber later development; (ii) “cellulose biosynthetic process” (GO:0030244, FDR = 9.9E-4), contributing to forming the major component of SCW; (iii) “glucuronoxylan biosynthetic process” (GO:0010417, FDR = 4.1E-3), containing important glycosyltransferase genes required for normal SCW biosynthesis [25-27]; (iv) “cellular cell wall organization” (GO:0007047, FDR = 2.4E-2), with genes encoding SCW-related cellulose synthase [37]. In addition, other important functional categories included “proton transport” (GO:0015992, FDR = 1.4E-2) and “establishment or maintenance of transmembrane electrochemical gradient” (GO:0010248, FDR = 5.4E-2), comprised of genes for H^+^-ATPase, proton pump interactor and vacuolar H^+^-translocating inorganic pyrophosphatase, which are important for regulation of electrochemical proton gradient and osmotic potential in the cell and also exert effects on plant growth and development [38,39].

At 19 DPA, up-regulated DEGs in TM-1 were associated with some of the same over-represented categories which were enriched at 16 DPA (Table 3). Aside from these common GO terms, other over-represented GO terms at 19 DPA included (i) “sucrose metabolic process” (GO:0005985, FDR = 2.8E-2), containing the genes encoding sucrose synthase, a key enzyme for fiber development, which degrades sucrose to supply UDPG as a substrate for cellulose biosynthesis [40]; and (ii) “cell wall thickening” (GO:0052386, FDR = 3.7E-2), containing the gene encoding l,3-β-glucanase, which is expressed at a low mRNA level in the elongation fibers but at a high mRNA level during SCW biosynthesis [41,42].

At 22 DPA, none of the GO terms were over-represented, but genes for putatively important seed storage proteins vicilin and legumin [43] were significantly up-regulated in TM-1 with 25.8 and 15.7-fold changes, respectively, implying putative inanition in *im* mutant during fiber development.

A comparison between the two lines at 25 DPA, the rapid SCW thickening stage, showed “response to chitin” (GO:0010200, FDR = 5.2E-28), “response to hormone stimulus” (GO:0009725, FDR = 5.2E-3) and “transcription, DNA-dependent” (GO:0006351, FDR = 2.5E-3) to be over-represented in TM-1. Most DEGs related to these GO terms encode TFs, including AP2/EREBP, NAC, zinc finger and WRKY proteins. The differential expression of these regulatory elements between TM-1 and *im* mutant emphasize the importance of this time point (25 DPA) in fiber SCW development.

### Carbohydrate active enzymes are involved in SCW biogenesis

**Figure 5:**
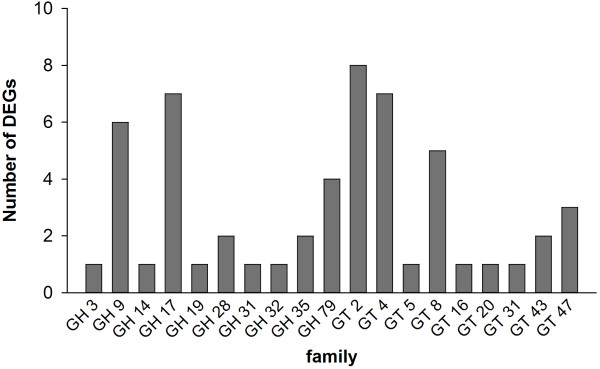
**Carbohydrate active enzyme family genes differentially expressed between TM-1 and *im *mutant.** GT, glycosyltransferase; GH, glycoside hydrolase.

Many over-represented GO terms of DEGs between TM-1 and *im* mutant were found to be related to cell wall biosynthesis and carbohydrate metabolism. Of them, most DEGs involved in these GO terms encode carbohydrate active enzymes. So we classified these DEGs into corresponding carbohydrate active enzymes families based on the Carbohydrate-Active Enzyme (CAZy) database [44] (http://www.cazy.org/) (Figure 5). A total of 55 DEGs belonged to 19 different glycosyltransferase (GT) and glycoside hydrolase (GH) families. Among GTs, the GT2 family contains cotton GhCesA2 and GhCesA5, which are similar to *Arabidopsis* CesA4 and CesA7, respectively, two SCW-relative cellulose synthases [37]. The GT4 family contains several different isoforms of sucrose synthase, which has been demonstrated to be an integral component of the cellulose synthase rosette [45]. Putative homologues of *Arabidopsis PARVUS*, *IRX9*, *IRX14*, *IRX7/FRA8* and *IRX10/GUT2* genes encode glycosyltransferases that belong to corresponding GT8, GT43 and GT47 families and regulate biosynthesis of polysaccharides matrix to affect SCW integrity [24-27,46]. Among GHs, endo-1,4-beta-glucanase belong to GH9, which is required for cellulose deposition. It presumably removes the primer from the growing cellulose chain, cleaves glucan chains to release tensional stress of cellulose crystallization or releases newly synthesized cellulose fibrils [47]. 1,3-beta-glucanase belongs to the GH17 family and has been proposed to degrade callose, which is deposited at the fiber base, and to open the plasmodesmta to promote transition from fiber elongation to SCW biosynthesis [42]. Beta-xylosidase belongs to the GH3 family and was reported to be involved in SCW metabolism and plant development [48]. Chitinase is a member of the GH19 family. We identified a chitinase-like protein gene (*GhCTL2*) in the microarray analysis, which was proposed to be expressed preferentially in cotton cells with secondary walls [29]. Interestingly, all of these genes had higher transcript levels in TM-1 than in the *im* mutant at 16 and/or 19 DPA, suggesting that they have roles in regulating the onset stage of SCW biosynthesis of cotton fiber.

### Confirmation of differential gene expression using quantitative real-time PCR (qRT-PCR)

**Figure 6:**
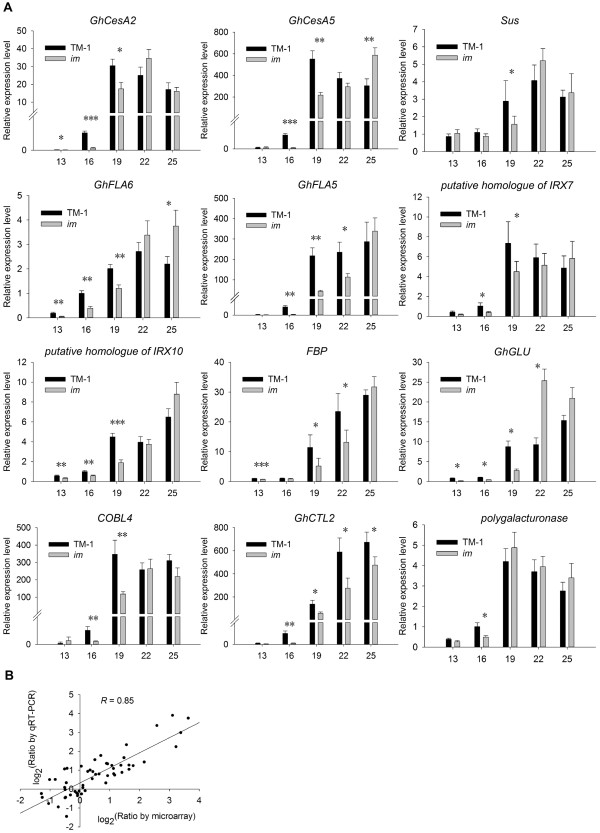
**qRT-PCR validation of selected DEGs. (A)** The x-axis represents five developmental periods, and the y-axis indicates the relative expression value by qRT-PCR analysis. Data shown are means of three biological replicates. Error bars indicate ± SD. **(B)** Comparison of gene expression ratios observed by microarray and qRT-PCR analyses. Data are from 12 probe sets at five time points between TM-1 and the *im* mutant. The microarray log_2_^(expression ratio)^ values (x-axis) are plotted against the log_2_^(expression ratio)^ obtained by qRT-PCR (y-axis). Asterisks indicate significant differences of expressional level (Student’s *t* test, *P < 0.05, **P < 0.01, ***P < 0.001) between TM-1 and the *im* mutant at same developmental stage.

Based on enrichment analysis of biological processes and expression profiles of genes differentially expressed during the transition and SCW biosynthesis stages of fiber development between TM-1 and *im* mutant, a total of 12 genes were selected for verification of the microarray data by qRT-PCR (Figure 6A). Although the microarray data at certain fiber developmental points were not supported well by qRT-PCR analysis, which may be partially due to cross-hybridization on the array with other family genes with similar sequences, most detected genes showed the same expression tendency by both methods. Overall, results of qRT-PCR were in agreement with the chip data with a high correlation coefficient (*R* = 0.85) (Figure 6B), supporting the validity of the microarray data in this study.

## Discussion

### The *Im* gene plays a key role in fiber development

The *im* mutant was identified as a mutant defective in fiber SCW development, which is due to the mutation of the *Im* gene [4]. It also had great influence on multiple fiber quality and yield traits, especially fiber maturity, micronaire and lint percentage affected by fiber SCW development [7,8]. In this study, the thickness of fiber SCW in the *im* mutant was reduced by about 20% compared with that in TM-1, which may be attributed to a reduction in deposition of cellulose, the main cell wall component in mature fibers. As an important indirect substrate for cellulose synthesis, the sucrose content in fibers of the mutant also was less than that in fibers of TM-1 during the rapid cellulose synthesis stage. The mutation of the *Im* gene was presumed to cause abnormal carbohydrate metabolism and a reduced amount of sucrose entering the cellulose synthesis pathway. As a result, the *im* mutant had low cellulose content, thin cell walls, as well as poor fiber fineness, fiber maturity and lint percentage.

### Asynchronous fiber developmental program in TM-1 and *im* mutant

During fiber development, fiber elongation and SCW biosynthesis overlap by several days. The onset of SCW biosynthesis typically occurs from 16–21 DPA [1,2]. In this study, we explored global patterns of gene expression in developing fibers of the *im* mutant and its NIL, TM-1, by the microarray method and revealed transcriptomic changes both within and between the two lines at different fiber developmental stages. The distribution of DEGs and over-represented GO terms within TM-1 and *im* mutant provided indications that fibers in TM-1 began to form SCW approximately from 16 DPA, whereas the same fiber developmental program in *im* was delayed until 19 DPA. Expression data from both microarray and qRT-PCR analyses showed higher transcript levels for some important SCW-related genes in TM-1 at 16 DPA. Significantly fewer transcriptomic changes were observed between TM-1 and the *im* mutant at 13 DPA, the elongation stage of fiber development, compared with the large number of DEGs between the two lines at 16 and 19 DPA, the onset of SCW biosynthesis. The asynchronous fiber developmental program in TM-1 and the *im* mutant suggests a defect in fiber development caused by the mutation of the *Im* gene.

### Carbohydrate metabolism and SCW biosynthesis

Secondary wall development of cotton fibers continues for a very long period of time, and cellulose synthesis and SCW thickening are the most important events during this developmental stage with many carbohydrate-active enzymes involved in this complex process. Pear et al. [28] first reported two cotton cellulose synthase genes, *GhCesA1* and *GhCesA2*, responsible for the SCW thickening of fibers. *GhCesA5* is similar to the *Arabidopsis* SCW-related gene *AtCesA7* and was reported to be co-expressed with *GhCesA1* and *GhCesA2* during cotton fiber secondary wall deposition [49]. In the present study, *GhCesA2* and *GhCesA5* shared similar expression patterns and exhibited several fold higher expression levels in TM-1 at the onset of SCW biosynthesis, suggesting a difference in cellulose synthesis between the two lines. During fiber development, approximately 80% of total carbon entering the fibers is converted to cellulose during the peak secondary wall synthesis stage, and much of the imported carbon is mainly in the form of sucrose [50]. Sucrose synthase (Sus) cleaves sucrose and supplies UDPG as a substrate for cellulose biosynthesis. It plays a critical role of Sus in channeling UDPG to the cellulose synthase [45]. In our microarray experiment, three cotton *Sus* genes were highly expressed and coordinately up-regulated with two cellulose synthase genes, *GhCesA2* and *GhCesA5*, during SCW thickening in TM-1 compared with that in the *im* mutant. The result is consistent with another report in poplar [51]. Fructose-1,6-bisphosphatase (FBP) is regarded as a key enzyme in the sucrose biosynthetic pathway [52]. Sharkey et al. [53] reported that conversion of carbon into sucrose is significantly reduced in *Flaveria linearis* mutants lacking cytosolic FBP activity. A decrease in the expression of *FBP* in *Solanum tuberosum*[54], *Arabidopsis thaliana*[55] and *Oryza sativa*[56] also was shown to lead to a reduction of sucrose synthesis. In the current study, *FBP* showed a higher expression level in TM-1 than in *im* mutant at the early stage of SCW biosynthesis, suggesting that more sucrose was synthesized earlier in TM-1 for supporting cellulose biosynthesis. In addition, the 1,3-beta-glucanase mRNA level was low in the elongation fibers but high during SCW biosynthesis [41]. Ruan et al. [42] proposed its role in fiber development, which may degrade callose deposition at the fiber base and open the plasmodesmta to promote the transition from fiber elongation to SCW biosynthesis. *GhCTL2* encoding a chitinase-like protein is known to be abundantly expressed during SCW biosynthesis [29], but the mechanism by which it acts is unclear.

In *Arabidopsis*, many irregular xylem mutants show defects in the development of secondary walls caused by loss of function of important carbohydrate active enzyme genes. In this study, we identified several orthologous genes of *Arabidopsis IRX* genes required for normal development of SCW, including *IRX7/FAR8*, *IRX9* and *IRX10/GUT2*[25,27,57]. They showed similar expression patterns and were up-regulated in TM-1 at 16 and/or 19 DPA, compared with *im* mutant, suggesting a putative variation in noncellulosic polysaccharide matrix between the two lines.

Arabinogalactan proteins (AGPs) are a kind of structural protein in plant cell wall. Fasciclin-like arabinogalactan proteins (FLAs) comprise one of the subclasses of AGPs [58]. Many studies have shown that FLAs are closely linked to SCW biosynthesis in different plants, including *Arabidopsis*, *Eucalyptus*, *Populus* and *Zinnia elegans*[59-61]. *GhFLA5* and *GhFLA6* are orthologous genes of *AtFLA12* and *AtFLA11* in cotton, respectively. Knockout of *AtFLA11* and *AtFLA12* has been shown to reduce cellulose and lignin content and affect stem tensile strength and stiffness, suggesting that *GhFLA5* and *GhFLA6* have similar functions as their counterparts in *Arabidopsis*[61]. By microarray analysis, we identified an orthologous gene of *Arabidopsis COBRA-like 4 (AtCOBL4)* which encodes a glycosylphosphatidylinositol (GPI)-anchored protein and is co-expressed with SCW-related *CesA* genes during SCW biosynthesis in *Arabidopsis*[22,23]. Its transcript abundance was approximately 9-fold higher in TM-1 than in *im* mutant at 16 DPA. Although the effect of this orthologous gene of *AtCOBL4* on fiber development is unknown, studies of the maize and rice orthologs of *AtCOBL4* suggest that it may be required for regulating deposition of SCW components [62,63].

### Toward mining *Im* candidate genes

In the comparative analysis of transcript profiling between TM-1 and the *im* mutant during fiber SCW development, a large number of DEGs were detected. Aside from the many genes involved in cellulose synthesis and SCW development, some genes, including *PARVUS*, *IRX7/FRA8*, *IRX9* and *IRX10* that participate in biosynthesis of hemicelluloses polysaccharides matrix and are necessary for normal development of xylem in *Arabidopsis*, were also found to be differentially expressed between TM-1 and the *im* mutant [64]. Although the role of these genes in SCW development of cotton fibers is unknown, these results suggest that the mutation of the *Im* gene caused variations of multiple cell wall components. We presumed that the *Im* gene may be an important regulatory element affecting fiber SCW development.

TFs are regulators that play key roles in plant development. However, in differentially expressed TF genes, we didn’t detect some TFs such as NAC, MYB and WRKY TFs reported to play important roles in regulating SCW development in previous studies [65,66]. One explanation is that the transcript encoding TF may not have been included in the microarray probe sets, or alternatively, it has very low abundance. Therefore, map-based cloning of *Im* should be conducted for cloning this important gene, elucidating its molecular mechanism and further genetic manipulation to improve fiber fineness and maturity.

Recently, information on the cotton D genome was published by Paterson et al. [67]. Using this information as a reference for mapping of the *Im* gene, molecular markers linked to the *im* locus on the A3 chromosome in a previous study [7] were projected on the D3 chromosome scaffold by custom perl scripts, based on the homology between the two chromosomes. We found that the positions of these molecular markers on the linkage map were highly consistent with those on the physical scaffold (Additional file 3), and a total of 155 genes were predicted in the physical interval on D3 scaffold based on the gene prediction result of D genome published on Phytozome website (http://www.phytozome.net/cotton.php) (Additional file 4). Of those, 8 genes included in the microarray analysis showed no differential expression between TM-1 and the *im* mutant. GO analysis showed 23 genes involved in important biological processes, including “signaling”, “biological regulation”, “growth”, “developmental process”, “microtubule-based process” and “carbohydrate metabolic process” (Additional file 5). The functional roles of these candidate genes during fiber development and the *Im* gene will be confirmed by experimental validation in future studies.

## Conclusions

The immature fiber (*im*) mutant can be regarded as an ideal research material for exploring fiber secondary cell wall (SCW) biogenesis. From the study, we confirmed that the significant differences between TM-1 and *im* mutant in cellulose content, thickness of cell wall and sucrose content, with asynchronous fiber developmental program in fiber SCW process. We propose that *Im* is an important regulatory gene influencing temporal differences in expression of genes related to fiber SCW biosynthesis. This study contributes to an increased insight for elucidating molecular mechanism of fiber SCW development.

## Methods

### Plant materials

Upland cotton genetic standard line Texas Marker-1 (*G. hirsutum* acc. TM-1) and the *im* mutant with a TM-1 background [4] were kindly provided by Dr. Russell J. Kohel (United States Department of Agriculture, Agricultural Research Service, College Station, TX). After introduction, the plants were selfed and developed. The two NILs were planted in the experimental field (Nanjing, China) using general farming practices in 2008. Flowers were hand-pollinated and tagged on the day of anthesis. For each fiber developmental stage, three biological pool samples, each with ten plants, were tested. Developing bolls were collected at 9 am at 3-day intervals from 13 through 25 DPA, and fibers were dissected from the cotton bolls immediately, frozen in liquid nitrogen and stored at −80°C.

### Cellulose content analysis

Cellulose content in fibers of TM-1 and the *im* mutant at 19, 22, 25, 30, 35 and 40 DPA and maturation fiber developmental stages was detected by using the FOSS Fibertec™ 1020 system (FOSS Inc., Hillerød, Denmark). Each sample had three biological replicates.

### Soluble carbohydrate analysis

The method for analyzing carbohydrate deposition was performed as described by Ruan et al. [68]. Approximately 0.2 g of fiber was ground in liquid nitrogen. The samples were extracted with 4 mL of preheated 80% ethanol for 5 min at 80°C. After cooling, the samples were centrifuged at 12,000 × *g* for 10 min. The supernatants were collected, while the pellets were resuspended in 2 mL of 50% ethanol and centrifuged again as described above. The resulting pellet was re-extracted with 2 mL of water and re-centrifuged. The total 8 mL of supernatant collected was mixed with an equal volume of chloroform and shaken vigorously. The aqueous phase was collected, dried in a vacuum and redissolved in 1 mL of water. The sucrose, glucose and fructose contents were measured and analyzed using an anion-exchange HPLC system (Agilent 1100 series; Agilent technologies, Inc., Santa Clara, CA, USA), which consisted of a G1311A pump and a G1362A refraction index detector. Sugar compounds were separated on a Sugar-D column (4.6 × 250 mm, Nacalai Tesque Inc., Kyoto, Japan), using acetonitrile/water (75: 25, v/v) as the mobile phase at the flow rate of 1.0 mL/min. The injection volume was 40 μL. Quantification of each sugar was accomplished by comparing the peak areas of the samples with those of standard solutions.

### Measurement of cell wall thickness of fibers on semi-thin sections

Cotton fibers at 13, 19, 25 DPA and maturation stage were used for statistical analysis of the cell wall thickness on semi-thin sections. The cotton fibers were fixed for 12 h in 2.5% glutaraldehyde. After three washes with 0.2 M PBS buffer, the fibers were dehydrated in a series of ethanol (50–100%) and epoxy ethane and then embedded in Epon resin for 48 h at 60°C. The middle parts of the fibers were cut into thin sections (3 μm) on an LKBIII Ultratome, and then the resin was dissolved very slightly using NaOH. The slices were placed on glass slides and examined under a microscope (Olympus BX 51, Tokyo, Japan). The thickness of the cell wall was evaluated using the Image-pro Plus program (Olympus). Approximately 300 fibers were measured for each sample.

### RNA isolation, amplification and labeling

Total RNA samples at different fiber developmental stages from 13–25 DPA in TM-1 and the *im* mutant were extracted using the CTAB-sour phenol extraction method [69]. The isolated RNA was purified using the NucleoSpin® RNA clean-up kit (MACHEREY-NAGEL, Düren, Germany) and checked for quantity and integrity by spectrophotometry and 1.2% agarose formaldehyde denaturing gel electrophoresis. RNA amplification and labeling were performed using the cRNA Amplification and Labeling Kit (CapitalBio, Beijing, China) per the manufacturer’s protocol. Briefly, total RNA was reversely transcribed into double-stranded cDNA using the CbcScript enzyme. Subsequently, cRNA was synthesized from cDNA products using the T7 Enzyme Mix in the cRNA Amplification and Labeling Kit and purified using the NucleoSpin® RNA clean-up kit. After the cRNA was synthesized, the new cDNA was generated with random primers from the cRNA using CbcScript II enzyme in the kit. Finally, the transcription products were purified with the Nucleospin® Extract II Kit (MACHEREY-NAGEL). The cDNA sample from TM-1 and *im* mutant at the same fiber developmental stage was labeled with Cy5 and Cy3-dCTP, respectively. And Cy5 and Cy3-dCTP interchanged to be used to label cDNA samples in the dye swap experiment.

### Microarray hybridization and statistical analysis

The dual-dye 28 k cotton cDNA microarray platform (GPL8569), containing 29,184 probes from −3 to 25 DPA Upland cotton (*G. hirsutum* L. cv. Xuzhou 142) ovules and fibers, was used to analyze expression patterns. All microarray data have been deposited in Gene Expression Omnibus of NCBI (GEO series accession number GSE49617, http://www.ncbi.nlm.nih.gov/geo/query/acc.cgi?acc=GSE49617). Cy3 or Cy5-labeled cDNAs were dissolved in 80 μl of hybridization solution containing 3× SSC, 0.2% SDS, 5× Denhardt’s solution and 25% formamide. DNA was denatured in the hybridization solution at 95°C for 3 min prior to loading onto a microarray. Arrays were hybridized at 42°C overnight and washed with two consecutive solutions (0.2% SDS, 2× SSC at 42°C for 5 min, and 0.2× SSC for 5 min at room temperature). The experimental design involved pairwise hybridizations between the two lines (TM-1 and *im* mutant), with two biological replicates and a dye swap, for fiber samples at 13, 16, 19, 22 and 25 DPA. All microarrays were scanned with a LuxScan 10 K/A scanner (CapitalBio) and array images were analyzed with LuxScan™ 3.0 (CapitalBio).

Statistical analyses were carried out using the LIMMA program [70] based on the open source Bioconductor project (http://www.bioconductor.org) in R. Faint spots with intensity < 400 units after subtraction of the background were removed. Subsequently, the data were normalized within arrays using the “LOWESS” method [71] and across arrays by the “Aquantile” method [72]. Normalized data were fitted to linear models in LIMMA to analyze DEGs using a moderated t-statistic (eBays) method [73]. DEGs between the two accessions at each time point were determined from the hybridization signal ratios on each slide, while the differential across time within each accession was determined from *in silico* comparisons of single channel data as indicated. Genes with FDR < 0.05 and a fold change ≥ 2 were identified as DEGs. GO analysis was performed for functional categorization of DEGs using Blast2GO software (http://www.blast2go.com/) [74]. Blast2GO includes GO annotation of DEGs and the function enrichment analysis for statistical assessment of annotation differences between two sets of sequences, using Fisher’s exact test for each GO term. FDR controlled *P* values [75] (FDR < 0.05) were used for the assessment of over-represented GO terms.

### qRT-PCR analysis

First-strand cDNA was synthesized in a final reaction volume of 25 μl containing 2 μg of RNA, 0.5 μg oligo(dT), 200 units M-MLV RT (Promega, Madison, WI, USA; Cat# M1705), 1.25 μl dNTP (10 mM) and 25 units RNasin Ribonuclease Inhibitor (Promega, Cat# N2511) according to the manufacturer’s instructions. Gene-specific qRT-PCR primers were synthesized commercially (Genscript, Nanjing, China) as listed in Additional file 6. qRT-PCR was performed using the LightCycler FastStart DNA Master SYBR Green I kit (Roche, Basel, Swiss) in AB7500 Real-Time PCR detection system (Applied Biosystems, Carlsbad, CA, USA). The qRT-PCR cycles were as follows: (1) 95°C, 10 min; (2) 95°C, 15 s; 60°C, 1 min for 40 cycles; (3) melting curve analysis from 65 to 95°C (1 s hold per 0.2°C increase) to check the specificity of the amplified product. The cotton *histone 3* gene (Acc. No. AF024716, forward primer: GGTGGTGTGAAGAAGCCTCAT, reverse primer: AATTTCACGAACAAGCCTCTGGAA) was used as the internal control to normalize the level of expression, and the amplification efficiency of each gene was calculated. The Pfaffl method was used to analyze expression data [76].

## Competing interests

The authors have declared that no competing interests exist.

## Authors’ contributions

Experiments were designed by WZG with suggestions from TZZ. Experiments were performed by CW. Bioinformatics analyses were performed by CW and YDL. WTX helped to carry out the soluble carbohydrate analysis. CW and WZG drafted the manuscript and WZG revised the manuscript. All authors read and approved the final manuscript.

## Supplementary Material

Additional file 1 **Catalog of all biological processes statistically over-represented during fiber developmental stages of TM-1 and *im *** **mutant.** Excel file containing the list of all over-represented (FDR < 0.05) biological processes during fiber developmental stages of TM-1 and *im* mutant. Click here for file

Additional file 2 **Complete list of developmental stage-specific biological processes over-represented between TM-1 and *im *** **mutant.** Excel file containing the list of all developmental stage-specific biological processes over-represented (FDR < 0.05) between TM-1 and *im* mutant. Click here for file

Additional file 3 **Projection of molecular markers of A3 linkage group containing *im *** **locus on D3 chromosome.** In TIF image file, A3 represents the linkage group containing the *im* locus constructed using *G. hirsutum* acc. *im* and *G. hirsutum* acc. CSIL028 F_2_ segregation population [7], D3 represent D3 chromosome in *G. raimondii*. Click here for file

Additional file 4 **Predicted 155 candidate *Im *** **genes.** Excel file containing predicted 155 candidate *Im* genes based on *im* mapping results and cotton D genome information published by Joint Genome Institute. Click here for file

Additional file 5 **List of 23 genes involved in important biological processes.** Excel file containing the list of 23 candidate *Im* gene involved in important biological processes including “signaling”, “biological regulation”, “growth”, “developmental process”, “microtubule-based process” and “carbohydrate metabolic process”. Click here for file

Additional file 6 **Gene-specific primers used in this study for qRT-PCR analysis.** Excel file containing all primer sequences used for the qRT-PCR experiment. Click here for file
