# Supplementary figures and images for: Aberrant phenotype and transcriptome expression during fiber cell wall thickening caused by the mutation of the Im gene in immature fiber (im) mutant in Gossypium hirsutum L
Source: BMC Genomics. 2014 Feb 1;15:94. doi: 10.1186/1471-2164-15-94 (PMC3925256; doi:10.1186/1471-2164-15-94)

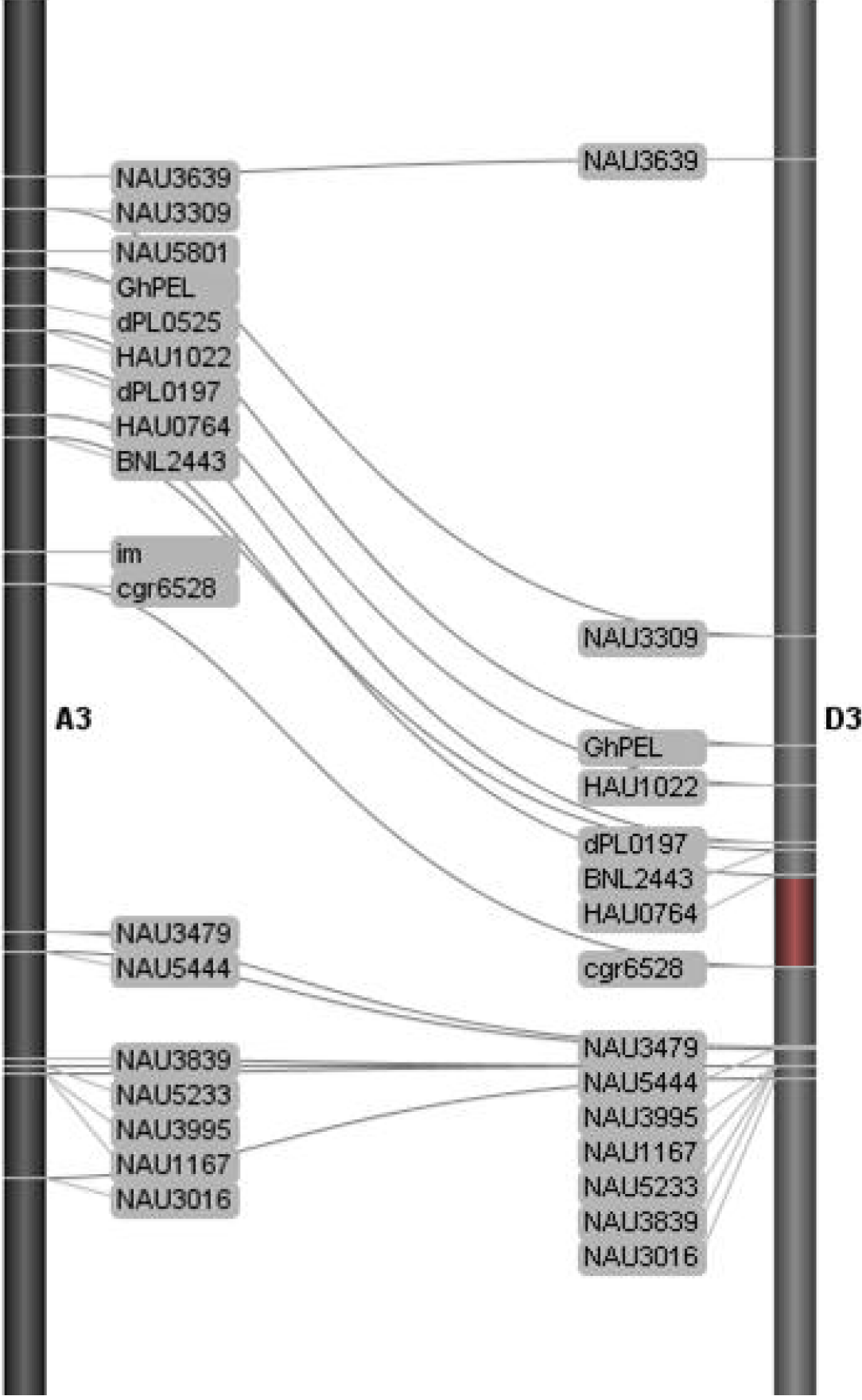

Supplement: Additional file 3 — Projection of molecular markers of A3 linkage group containing im locus on D3 chromosome. In TIF image file, A3 represents the linkage group containing the im locus constructed using G. hirsutum acc. im and G. hirsutum acc. CSIL028 F2 segregation population [7], D3 represent D3 chromosome in G. raimondii. [file 1471-2164-15-94-S3.tiff]
